# Association of renal hyperfiltration with incidence of dyslipidemia: a nationwide retrospective longitudinal cohort study

**DOI:** 10.1371/journal.pone.0324710

**Published:** 2025-06-03

**Authors:** Hyungjong Park, Min Kyoung Kang, Tae-Jin Song

**Affiliations:** 1 Department of Neurology, Keimyung University School of Medicine, Daegu, Republic of Korea; 2 Department of Neurology, Seoul Hospital, Ewha Womans University College of Medicine, Seoul, Republic of Korea; National Healthcare Group, SINGAPORE

## Abstract

**Background:**

Individuals with chronic kidney disease (CKD) are often diagnosed with dyslipidemia, contributing to their cardiovascular risk. However, the relationship between renal hyperfiltration (RHF) and dyslipidemia remains unclear. Here, we aimed to examine the association between RHF and incidence of dyslipidemia.

**Methods:**

We examined the data of 2,780,874 participants who underwent health examinations between 2010 and 2011 from the Korean NHIS-HEALS database. Renal function was assessed using estimated glomerular filtration rate (eGFR) levels that were calculated using the Chronic Kidney Disease Epidemiology Collaboration equation. The incidence of dyslipidemia was defined as the outcome. Dyslipidemia was characterized by meeting the following criteria: having at least one claim of International Classification of Diseases (ICD)-10 code (E78) with an anti-dyslipidemia agent prescription, claims of ICD-10 code (E78) occurring more than twice, a total cholesterol level ≥ 240 mg/dL, low-density lipoprotein cholesterol ≥ 160 mg/dL, triglycerides ≥ 200 mg/dL, or high-density lipoprotein cholesterol < 40 mg/dL. RHF was defined as having an eGFR > 120 mL/min ∙ 1.73 m^2^ defined by the eGFR range or being in the 10^th^ decile of whole eGFR level.

**Results:**

Over a median follow-up of 9.56 years, 2,243,502 participants with dyslipidemia were identified. The mean age of participants was 47.35 ± 13.96 years. In the multivariable Cox regression analysis, eGFR > 120 mL/min ∙ 1.73 m^2^ was associated with a decreased risk of dyslipidemia (HR: 0.35; 95% CI [0.34–0.36]). Furthermore, compared to the fifth decile, tenth (≥ 114.21) (HR: 0.47, 95% CI (0.46–0.48) eGFR deciles were significantly associated with a reduced incidence of dyslipidemia.

**Conclusions:**

The general population with RHF may have a lower risk of incidence of dyslipidemia. The identified inverse association between RHF and dyslipidemia underscores its significance in risk stratification and preventive interventions in clinical practice.

## Introduction

Renal function is associated with cardiovascular disease (CVD) and related risk factors, including myocardial infarction, stroke, atrial fibrillation, and heart failure. Chronic kidney disease (CKD) is defined as a decreased estimated glomerular filtration rate (eGFR), and reduced eGFR levels are significantly correlated with an increased likelihood of developing CVD [1,2].

In addition to a low eGFR, an unusually higher-than-normal eGFR, called renal hyperfiltration (RHF), can also be associated with various health issues. RHF is considered a regular physiological condition; however, it may signal an underlying abnormal kidney function. For example, it could indicate the initial signs of damage to the kidney’s filtering units in individuals with high blood pressure or imply the presence of preclinical kidney disease in those with diabetes [3]. Furthermore, RHF is strongly associated with various vascular risk factors, including hypertension, prediabetes, obesity, and obstructive sleep apnea, all contributing to cardiovascular or cerebrovascular events [4].

Dyslipidemia refers to elevated levels of lipids in the blood, particularly low-density lipoprotein cholesterol (LDL-c). It is a major risk factor for atherosclerotic CVD, including coronary heart disease, stroke, and peripheral arterial disease. Untreated or inadequately managed dyslipidemia can lead to fatal and nonfatal cardiovascular or cerebrovascular events [5–7]. In addition, dyslipidemia amplifies atherosclerosis in renal vessels, leading to further kidney dysfunction. Uremic toxins that reduce renal function exacerbate dyslipidemia [8].

Although RHF is often regarded as an indicator of good renal function, there is a possibility that individuals with RHF might exhibit a lower risk of developing dyslipidemia. Conversely, if RHF is an early sign of kidney disease, it could indicate an increased risk of dyslipidemia. However, research on the possible correlation between RHF and dyslipidemia development is limited. Therefore, this nationwide longitudinal study aimed to investigate the association between RHF and the risk of dyslipidemia in the general Korean population.

## Materials and methods

### Data source

Data were sourced from the NHIS-HEALS database, a subset of the Korean National Health Insurance Service. The NHIS is a government initiative that covers health insurance for approximately 97% of the Korean population. The remaining 3% not covered by the NHIS are covered by the Medical Aid program, also under NHIS supervision [9,10].

The NHIS encourages participants to undergo standardized health check-ups every 2 years to aid in the early identification and prevention of diseases. The NHIS-HEALS database collects various information, including demographic details, socioeconomic backgrounds, health screening results, recorded diagnoses, and treatment specifics. These screenings involve assessments such as height, weight, blood pressure measurements, laboratory tests, and evaluations of lifestyle behaviors [11–16].

### Study population

**Fig 1:**
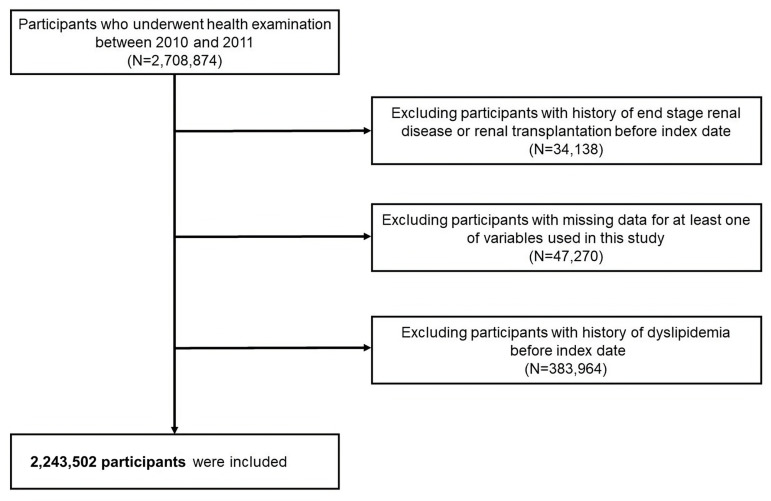
Flow chart depicting the selection process for study participants.

The study cohort from the NHIS-HEALS database included 2,708,874 individuals aged 20–79 years who participated in health screenings between 2010 and 2011 (under the dataset identifier NHIS-2022-01-313) [10,17,18]. We excluded individuals who had undergone renal transplantation or were diagnosed with end-stage renal disease before the study, totaling 34,138 individuals. Additionally, 47,270 participants were removed owing to incomplete data for the variables of interest. Furthermore, 383,964 individuals with a history of dyslipidemia before the study were excluded, resulting in a final sample of 2,243,502 participants for analysis (Fig 1).

### Definitions and variables

The starting point for tracking each participant’s outcome, referred to as the index date, was established based on the date of their health evaluation. To determine eGFR, serum creatinine levels from the health check-up were used along with the formulas provided by the Chronic Kidney Disease Epidemiology Collaboration (CKD-EPI) (S1 File) [19]. Baseline characteristics, including age, sex, body mass index (BMI), waist circumference, and household income, were assessed on the index date. Details on habits, such as smoking, alcohol consumption, and regular exercise, were collected through questionnaires. Smoking habits were classified as non-smoking, past smoking, or active smoking. Both alcohol intake and consistent physical activity were noted in terms of their frequency per week. Proteinuria was confirmed if the urine dipstick test showed a result of ≥ + 1. Comorbidities, including hypertension, diabetes mellitus, dyslipidemia, heart failure, myocardial infarction, valvular heart disease, and hyperthyroidism, were identified between January 2009 and the index date using specific criteria (S1 Table). Diagnostic codes were classified based on the International Classification of Diseases (ICD)-10 according to previous studies [20–25]. The primary outcome was the incidence of dyslipidemia, defined as having at least one claim of ICD-10 code (E78) with the prescription of an anti-dyslipidemia agent, claims of ICD-10 code (E78) more than twice, total cholesterol level ≥ 240 mg/dL, LDL-C ≥ 160 mg/dL, triglycerides ≥ 200 mg/dL, or high-density lipoprotein cholesterol < 40 mg/dL based on fact-sheets in Korean society of lipid and atherosclerosis [26].

### Statistical analysis

The data results were presented as means ± standard deviation or as numbers and percentages. The association between eGFR and the incidence of dyslipidemia was investigated by categorizing participants into eGFR range (< 60, 60–89, 90–120, and > 120 mL/min ∙ 1.73 m^2^), based on guidelines and previous literature [1,4]. Additionally, to examine trends across the different eGFR levels, participants were stratified into deciles. Participants with eGFR > 120 mL/min ∙ 1.73 m^2^ and those in the 10^th^ decile (114.12 mL/min ∙ 1.73 m^2^) was designated as the RHF group. The reference group for comparison were the range of 60–89 mL/min ∙ 1.73 m^2^ and the fifth decile of 87.12–91.22 mL/min ∙ 1.73 m^2^. Kaplan–Meier survival curves were used to analyze this relationship, and log-rank tests were employed to compare the differences between eGFR range and deciles. Cox proportional hazard models were used to calculate hazard ratios (HR) and 95% confidence intervals (CI) to determine the relationship between eGFR and the incidence of dyslipidemia. Multivariable regression models were applied to account for potential confounding variables, including sex, age, body mass index, waist circumference, income level, smoking, alcohol consumption, regular physical activity, proteinuria, hypertension, diabetes mellitus, heart failure, myocardial infarction, hyperthyroidism, and the Charlson comorbidity Index. Subgroup analyses were conducted considering age and body mass, which are closely related to eGFR. However, further evaluation was performed for the sensitivity analysis using eGFR levels according to the Modification of Diet in Renal Disease (MDRD) study equation [27]. Statistical analyses were conducted using SAS software (version 9.2, SAS Institute, Cary, NC, USA), with a *p*-value of less than 0.05 considered significant.

### Ethical approval statement

This study was approved by the Institutional Review Board of the Ewha Womans University College of Medicine, which waived the requirement for informed consent (approval number: SEUMC 2023-05-020). This is because the NHIS allows researchers to restrict the use of anonymous data for research objectives. Additionally, our manuscript follows research and publication ethics and has not been published elsewhere.

## Results

The average age of the participants was 47.35 ± 13.96 years, with males comprising 52.01% of the population. The prevalence rates of current smoking, hypertension, and diabetes mellitus were 24.59%, 13.31%, and 5.05%, respectively. Regarding the eGFR deciles, 10.0% of participants were in the 5^th^ group (reference), corresponding to an eGFR of 87.12–91.22 mL/min ∙ 1.73 m^2^. The proportions of participants with eGFR levels < 60, 60–89 (reference), 90–120, and > 120 mL/min ∙ 1.73 m^2^ were 3.59%, 43.48%, 48.28%, and 4.64%, respectively (Table 1).

**Table 1:** Baseline characteristics of participants.

Variables	Total (n = 2,243,502)
Sex	
Male	1,166,923 (52.01)
Female	1,076,579 (47.99)
Age, years	47.35 ± 13.96
Body mass index (kg/m^2^)	23.57 ± 3.24
Waist circumference (cm)	79.70 ± 9.29
Household income	
Q1, lowest	615,660 (27.44)
Q2	798,228 (35.55)
Q3	556,695 (24.81)
Q4, highest	272,919 (12.16)
Smoking status	
Never	1,372,302 (61.17)
Former	319,458 (14.24)
Current	551,742 (24.59)
Alcohol consumption (days/week)	
None	1,162,580 (51.82)
1–4	993,537 (44.29)
≥5	87,385 (3.90)
Regular physical activity (days/week)	
None	1,371,539 (61.13)
1–4	210,270 (9.37)
≥5	661,693 (29.49)
Proteinuria	
Negative (-)	2,150,343 (95.85)
Positive (+)	93,159 (4.15)
Total cholesterol (mg/dL)	199.93 ± 29.32
Comorbidities	
Diabetes mellitus	113,378 (5.05)
Hypertension	298,703 (13.31)
Heart failure	21,284 (0.95)
Myocardial Infarction	3,555 (0.16)
Valvular heart disease	4,724 (0.21)
Cardiomyopathy	1,346 (0.05)
Hyperthyroidism	20,777 (0.93)
Congenital heart disease	661 (0.03)
Charlson comorbidity index	
0	1,460,775 (65.11)
1	490,581 (21.87)
≥2	292,146 (13.02)
eGFR (range), mL/min/1.73 m^2^	
< 60	80,558 (3.59)
60–89	975,566 (43.48)
90–120	1,083,266 (48.28)
> 120	104,112 (4.64)
eGFR (decile), mL/min/1.73 m^2^	
1st (< 68.61)	224,820 (10.02)
2nd (68.62–76.14)	222,828 (9.93)
3rd (76.24–82.03)	225,428 (10.05)
4th (82.09–87.02)	222,075 (9.90)
5th (87.12–91.22)	227,474 (10.14)
6th (91.40–96.49)	226,781 (10.11)
7th (96.69–101.26)	223,795 (9.98)
8th (101.27–106.75)	219,073 (9.76)
9th (106.86–114.12)	224,665 (10.01)
10th (≥ 114.21)	226,563 (10.10)

Data are presented as the mean ± standard deviation, or number (percentage)

Q, quartile; eGFR, estimated glomerular filtration rate.

Among the four groups categorized by eGFR range, the group with a higher eGFR range was associated with younger age, lower body mass index, smaller waist circumference, and a lower proportion of individuals with hypertension, diabetes, and a Charlson comorbidity index of 2 or higher (Table 2).

**Table 2:** Baseline characteristics of participants by the eGFR range.

Variables	Total (n = 2,243,502)	< 60 (n = 80,558)	60-89 (n = 975,566)	90-120 (n = 1,083,266)	> 120 (n = 104,112)	P-value
Sex						<.001
Male	1,166,923 (52.01)	34,679 (43.05)	526,667 (53.99)	567,061 (52.35)	38,516 (36.99)	
Female	1,076,579 (47.99)	45,879 (56.95)	448,899 (46.01)	516,205 (47.65)	65,596 (63.01)	
Age, years	47.35 ± 13.96	66.55 ± 11.97	51.75 ± 13.48	43.60 ± 11.69	30.34 ± 7.96	<.001
Body mass index (kg/m^2^)	23.57 ± 3.24	24.14 ± 3.24	23.82 ± 3.13	23.42 ± 3.58	22.27 ± 3.32	<.001
Waist circumference (cm)	79.7 ± 9.29	82.82 ± 9.29	80.65 ± 9.06	79.06 ± 10.22	74.99 ± 8.93	<.001
Household income						<.001
Q1, lowest	615,660 (27.44)	21,598 (26.81)	251,580 (25.79)	302,225 (27.90)	40,257 (38.67)	
Q2	798,228 (35.58)	23,319 (28.95)	320,538 (32.86)	408,165 (37.68)	46,206 (44.38)	
Q3	556,695 (24.81)	21,535 (26.73)	260,158 (26.67)	260,806 (24.08)	14,196 (13.64)	
Q4, highest	272,919 (12.16)	14,106 (17.51)	143,290 (14.68)	112,070 (10.34)	3,453 (3.31)	
Smoking status						<.001
Never	1,372,302 (61.17)	58,000 (72.00)	595,166 (61.01)	647,210 (59.75)	71,926 (69.09)	
Former	319,458 (14.24)	12,419 (15.42)	159,755 (16.37)	139,639 (12.89)	7,645 (7.34)	
Current	551,742 (24.59)	10,139 (12.58)	220,645 (22.62)	296,417 (27.36)	24,541 (23.57)	
Alcohol consumption (days/week)						<.001
None	1,162,580 (51.82)	60,203 (74.73)	533,963 (54.73)	521,999 (48.19)	46,415 (44.58)	
1–4	993,537 (44.29)	17,176 (21.32)	400,196 (41.02)	520,507 (48.05)	55,658 (53.46)	
≥5	87,385 (3.90)	3,179 (3.95)	41,407 (4.25)	40,760 (3.76)	2,039 (1.96)	
Regular physical activity (days/week)					104112	<.001
None	1,371,539 (61.13)	56,859 (70.58)	587,272 (60.20)	659,781 (60.91)	67,627 (64.96)	
1–4	210,270 (9.37)	6,424 (7.97)	98,472 (10.09)	98,314 (9.08)	7,060 (6.78)	
≥5	661,693 (29.49)	17,275 (21.45)	289,822 (29.71)	325,171 (30.01)	29,425 (28.26)	
Proteinuria						<.001
Negative (-)	2,150,343 (95.85)	72,058 (89.45)	934,059 (95.75)	1,044,074 (96.38)	100,152 (96.20)	
Positive (+)	93,159 (4.15)	8,500 (10.55)	41,507 (4.25)	39,192 (3.62)	3,960 (3.8)	
Total cholesterol (mg/dL)	199.93 ± 29.32	199.98 ± 32.14	199.82 ± 30.27	199.73 ± 31.17	199.18 ± 29.21	<.001
Comorbidities						
Diabetes mellitus	113,378 (5.05)	10,333 (12.83)	55,921 (5.72)	1,037,957 (95.82)	1,815 (1.74)	<.001
Hypertension	298,703 (13.31)	21,004 (26.07)	150,181 (15.39)	121,930 (11.26)	5,588 (5.36)	<.001
Heart failure	21,284 (0.95)	4,702 (5.84)	11,608 (1.19)	4,581 (0.42)	123 (0.12)	<.001
Myocardial infarction	3,555 (0.16)	648 (0.80)	1,956 (0.20)	915 (0.08)	36 (0.03)	<.001
Valvular heart disease	4,724 (0.21)	828 (1.03)	2,491 (0.26)	1,349 (0.12)	56 (0.05)	<.001
Cardiomyopathy	1,346 (0.06)	299 (0.37)	695 (0.07)	340 (0.03)	12 (0.01)	<.001
Hyperthyroidism	20,777 (0.93)	950 (1.18)	8,631 (0.88)	10,170 (0.94)	1,026 (0.99)	<.001
Congenital heart disease	661 (0.03)	42 (0.05)	286 (0.03)	298 (0.03)	35 (0.03)	<.001
Charlson comorbidity index						<.001
0	1,460,775 (65.11)	28,805 (35.76)	597,309 (61.22)	752,371 (69.45)	82,290 (79.04)	
1	490,581 (21.87)	20,770 (25.78)	226,581 (23.23)	225,668 (20.83)	17,562 (16.87)	
≥2	292,146 (13.02)	30,983 (38.46)	151,676 (15.55)	105,227 (9.72)	4,260 (4.09)	

Data is presented as the mean ± standard deviation, or as a number (percentage).

SD, standard deviation; Q, quartile; eGFR, estimated glomerular filtration rate.

**Fig 2:**
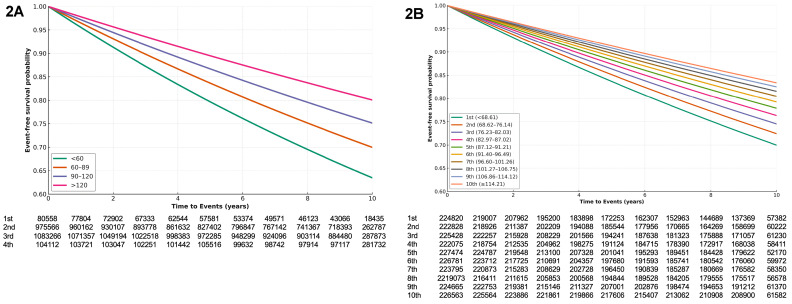
Kaplan–Meier survival curves illustrating the relationship between estimated glomerular filtration rate (eGFR) and incidence of dyslipidemia: (2A) eGFR ranges and (2B) eGFR deciles.

During a median follow-up of 9.56 years (interquartile range: 9.18–10.09 years), 430,980 cases of dyslipidemia (18.92%) were documented. Patients who had newly diagnosed dyslipidemia were older, a higher body mass index and had more comorbidities including diabetes mellitus, hypertension, heart failure and myocardial infarction (S2 Table). Kaplan–Meier survival analysis indicated that the risk of dyslipidemia was significantly associated with eGFR range (*p* < 0.001) and eGFR deciles (*p *< 0.001) (Fig 2A, B).

In the multivariable analysis, eGFR > 120 mL/min ∙ 1.73 m^2^ was associated with a decreased risk of dyslipidemia (HR: 0.35, 95% CI [0.34–0.36], p < 0.001). Additionally, compared to the 5^th^ decile, the 9^th^ (106.86–114.12 mL/min ∙ 1.73 m^2^) (HR: 0.82, 95% CI [0.81–0.83], p < 0.001) and 10^th^ (≥114.21) (HR: 0.47, 95% CI [0.46–0.48], p < 0.001) eGFR deciles were significantly associated with a reduced incidence of dyslipidemia (Table 3, S4 Table).

**Table 3:** Relation between eGFR levels (categorized by eGFR ranges and deciles) and the incidence of dyslipidemia.

	Number of participants	Number of events	Event rate (%) (95% CI)	Person-years	Incidence rate (per 1,000 person-years)	Adjusted HR (95% CI)	P-value
eGFR, mL/min/1.73 m^2^ (range)							
< 60	80,558	23,467	29.13 (28.82, 29.44)	587,215.93	39.96	1.02 (1.01, 1.04)	0.002
60–90	975,566	221,324	22.69 (22.60, 22.77)	8,160,156.62	27.12	Reference	
90–120	1,083,266	186,152	17.18 (17.11, 17.26)	9,466,641.71	19.66	0.83 (0.82, 0.84)	<.001
> 120	104,112	6,612	6.35 (6.20, 6.50)	966,545.11	6.84	0.35 (0.34, 0.37)	<.001
eGFR, mL/min/1.73 m^2^ (decile)							
1st (< 68.61)	224,820	64,062	28.49 (28.31, 28.68)	1,735,580.95	36.91	1.29 (1.27, 1.31)	<.001
2nd (68.62–76.14)	222,828	54,912	24.64 (24.46, 24.82)	1,837,642.05	29.88	1.23 (1.22, 1.25)	<.001
3rd (76.24–82.03)	225,428	48,525	21.53 (21.36, 21.70)	1,909,012.13	25.42	1.14 (1.12, 1.15)	<.001
4th (82.09–87.02)	222,075	46,889	21.11 (20.94, 21.28)	1,877,365.33	24.98	1.14 (1.11, 1.15)	<.001
5th (87.12–91.22)	227,474	42,016	18.47 (18.31, 18.63)	1,966,542.26	21.37	Reference	
6th (91.40–96.49)	226,781	45,825	20.21 (20.04, 20.37)	1,937,121.43	23.66	1.08 (1.06, 1.09)	<.001
7th (96.69–101.26)	223,795	44,525	19.90 (19.73, 20.06)	1,922,748.72	23.16	1.11 (1.09, 1.12)	<.001
8th (101.27–106.75)	219,073	41,640	19.01 (18.84, 19.17)	1,898,236.11	21.94	1.07 (1.05, 1.09)	<.001
9th (106.86–114.12)	224,665	32,332	14.39 (14.25, 14.54)	2,002,746.10	16.14	0.82 (0.80, 0.83)	<.001
10th (≥ 114.21)	226,563	16,829	7.43 (7.32, 7.54)	2,093,564.29	8.04	0.46 (0.45, 0.47)	<.001

The multivariable model was adjusted for sex, age, income levels, smoking, alcohol consumption, regular physical activity, body mass index, waist circumference, proteinuria, total cholesterol, hypertension, diabetes mellitus, heart failure, myocardial infarction, valvular heart disease, cardiomyopathy, hyperthyroidism, and Charlson comorbidity index.

HR, hazard ratio; CI, confidence interval; eGFR, estimated glomerular filtration rate.

**Fig 3:**
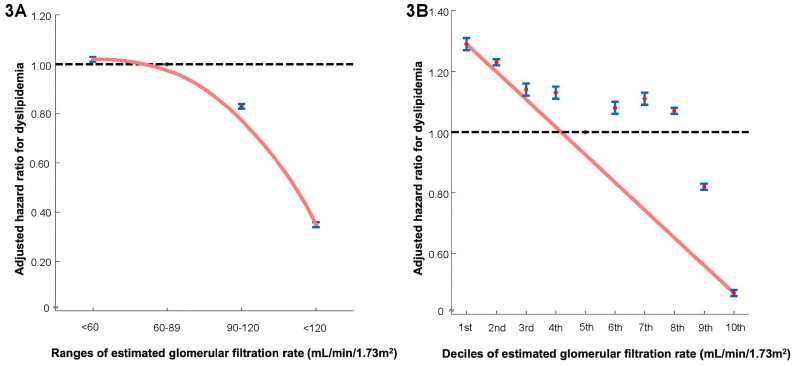
Hazard ratios for dyslipidemia based on estimated glomerular filtration rate: (3A) ranges, (3B) deciles. The hazard ratios representing the association between renal function and the incidence of dyslipidemia are displayed in two formats: (A) ranges and (B) deciles. The solid blue line shows the multivariate-adjusted hazard ratios with 95% confidence intervals for each group, while the dashed lines indicate a hazard ratio of 1. The red line represents the restricted cubic spline curves. Hazard ratios were calculated using the multivariable Cox after adjusting sex, age, income levels, smoking, alcohol consumption, regular physical activity, body mass index, waist circumference, proteinuria, total cholesterol, hypertension, diabetes mellitus, heart failure, myocardial infarction, hyperthyroidism, and Charlson comorbidity index.

The hazard ratio plot demonstrated a decline in the hazard ratio for dyslipidemia as the eGFR increased in both decile (Fig 3A) and ranges (Fig 3B).

These associations of RHF with the incidence of dyslipidemia, evaluated across both eGFR ranges and deciles, were consistently noted regardless of age groups (< 65 or ≥ 65 years) (S5 and S6 Tables). Similarly, significant associations remained consistent irrespective of BMI category (< 25 kg/m^2^ or ≥ 25 kg/m^2^) (S7 and S8 Tables), presence of diabetes (S9 and S10 Tables), or hypertension (S11 and S12 Tables). Furthermore, the association between eGFR and the incidence of dyslipidemia was consistently observed in the sensitivity analyses, regardless of whether eGFR was measured using the MDRD method (S13 Table).

## Discussion

The key findings of our study were that individuals with eGFR > 120 mL/min ∙ 1.73 m^2^ or the 10^th^ decile (≥ 114.21 ml/min ∙ 1.73 m²) exhibited a lower risk of dyslipidemia incidence. These findings were consistent regardless of age, obesity, the presence of diabetes mellitus or hypertension.

RHF, marked by an abnormally high eGFR, is characterized by an elevated filtration rate per nephron. RHF is known to manifest in various conditions, including physiological states such as pregnancy and pathological conditions such as a high-protein diet and obesity [3,4]. While the mechanism of RHF is not fully elucidated, one hypothesis indicates that an imbalance of vasoactive factors controlling pre-and post-glomerular arteriolar tone leads to preglomerular vasodilation and post-glomerular vasoconstriction, resulting in glomerular hyperfiltration [28]. Currently, RHF is recognized as a risk factor for the development of CVD, all-cause mortality, and clinical and subclinical CVD in patients with or without vascular disease [3]. However, in certain diseases, such as ischemic stroke, the influence of RHF on the development of ischemic stroke or outcome after ischemic stroke remains controversial [29]. Additionally, our study revealed that contrary to the prevailing notion that RHF negatively influences the development of dyslipidemia, its presence is associated with a reduced likelihood of developing dyslipidemia.

The protective effects of RHF against the development of dyslipidemia may be attributed to several mechanisms. Lipids are the primary sources of energy for kidney metabolism. In particular, mitochondrial oxidation of free fatty acids significantly contributes to ATP production in the kidney, particularly in the proximal tubules [30]. Therefore, elevated kidney metabolism during RHF leads to increased lipid consumption, potentially exerting a protective effect against dyslipidemia development. Secondly, chronic inflammation mediates preconditioning and protects against the development of severe events [31]. In RHF, low-grade systemic inflammation correlates with oxidative stress, potentially inducing damage to circulating creatinine levels. This may promote its aggregation with components such as apoA-1 and adiponectin, reducing serum creatinine levels and consequently causing RHF [4]. Therefore, individuals with RHF are assumed to be consistently exposed to chronic inflammation, which can promote preconditioning and potentially prevent dyslipidemia. Finally, certain physiological states or biological markers that initially appear benign or even protective in a general population may become risk factors after disease develops. High bone mineral density and increased heart rate variability are representative markers of this phenomenon [32,33]. This phenomenon, referred to as “threshold effects”, helps explain paradoxical observations of certain biomarkers [34]. In individuals with RHF, the condition exerts an effect analogous to that of biomarkers that exhibit threshold effects when the critical threshold maintained by renal reserves is exceeded. This threshold-based concept may also explain the protective association of dyslipidemia observed in our general cohort analysis.

Currently, there is no established standard cut-off value for defining RHF. Several studies applied a unified cut-off value for defining RHF, while other relied on the percentiles of eGFR [4,29]. In our analysis, we defined RHF using an eGFR > 120 ml/min/1.73m^2^ and 10th deciles of eGFR (114.21 ml/min/1.73m^2^). Thus, future studies should aim to identify a precise and widely accepted cut-off value for RHF.

Our study has several limitations that need to be recognized. First, the potential ethnic bias in our findings could limit the broader applicability to other ethnicities and raise concerns regarding the generalizability of our conclusions. Therefore, additional studies across various racial and ethnic groups should be conducted. Second, our study only examined eGFR in a cross-sectional manner at a single time point and did not consider temporal changes in the eGFR. Additionally, cystatin C, recognized as the best indicator of renal function, was not measured in the NHIS-HEAL dataset, posing a limitation to our study. Third, we may have overestimated the kidney function in individuals with an eGFR > 90 mL/min ∙ 1.73 m^2^, which might affect the reliability of our results. Fourth, baseline lipid parameters such as high-density lipoprotein, low-density lipoprotein, and triglycerides, which could also influence dyslipidemia development, were not collected. Instead, only total cholesterol levels were collected and adjusted for, and our results remained consistent despite this limitation. Finally, despite the comprehensiveness and longitudinal nature of our nationwide study, its retrospective design makes it challenging to determine cause-and-effect relationships.

## Conclusions

Our study demonstrated that RHF (eGFR > 120 mL/min/1.73 m^2^ or eGFR 10^th^ decile [>114.21 mL/min/1.73 m^2^]) was associated with a reduced risk of dyslipidemia. The general population with RHF may have a lower risk of incidence of dyslipidemia.

## Supporting information

S1 File Data availability statement. (DOCX)

S2 File Chronic Kidney Disease Epidemiology Collaboration (CKD-EPI) formula. (DOCX)

S1 Table Definition of covariates. (DOCX)

S2 Table Comparison of baseline characteristics according to the incidence of dyslipidemia. (DOCX)

S3 Table Association factors of renal hyperfiltration (range) with the incidence of dyslipidemia. (DOCX)

S4 Table Association factors of renal hyperfiltration (decile) with the incidence of dyslipidemia. (DOCX)

S5 Table Association of renal hyperfiltration (range) with the incidence of dyslipidemia according to age. (DOCX)

S6 Table Association of renal hyperfiltration (decile) with the incidence of dyslipidemia according to age. (DOCX)

S7 Table Association of renal hyperfiltration (range) with the incidence of dyslipidemia according to BMI. (DOCX)

S8 Table Association of renal hyperfiltration (decile) with the incidence of dyslipidemia according to BMI. (DOCX)

S9 Table Association of renal hyperfiltration (range) with the incidence of dyslipidemia according to diabetes mellitus. (DOCX)

S10 Table Association of renal hyperfiltration (decile) with the incidence of dyslipidemia according to diabetes mellitus. (DOCX)

S11 Table Association of renal hyperfiltration (range) with the incidence of dyslipidemia according to hypertension. (DOCX)

S12 Table Association of renal hyperfiltration (decile) with the incidence of dyslipidemia according to hypertension. (DOCX)

S13 Table Association of renal hyperfiltration (range and decile) with the incidence of dyslipidemia based on MDRD methods. (DOCX)
