# Supplementary material for: Association of renal hyperfiltration with incidence of dyslipidemia: a nationwide retrospective longitudinal cohort study
Source: PLoS One. 2025 Jun 3;20(6):e0324710. doi: 10.1371/journal.pone.0324710 (PMC12133170; doi:10.1371/journal.pone.0324710)
Supplement: S1 File — (DOCX) [file pone.0324710.s001.docx]

**Supplementary method 1.** Data availability statement.

The data used in this study are available in the National Health Insurance Service-National Health Screening Cohort (NHIS-HEALS) database, but restrictions apply to the public availability of these data used under license for the current study. Requests for access to the NHIS data can be made through the National Health Insurance Sharing Service homepage [http://nhiss.nhis.or.kr/bd/ab/bdaba021eng.do]. For access to the database, a completed application form, research proposal, and application for approval from the Institutional Review Board should be submitted to the inquiry committee of research support in the NHIS for review.
