# Supplementary material for: Association of renal hyperfiltration with incidence of dyslipidemia: a nationwide retrospective longitudinal cohort study
Source: PLoS One. 2025 Jun 3;20(6):e0324710. doi: 10.1371/journal.pone.0324710 (PMC12133170; doi:10.1371/journal.pone.0324710)
Supplement: S2 File — (DOCX) [file pone.0324710.s002.docx]

**Supplementary method 2.** Chronic Kidney Disease Epidemiology Collaboration (CKD-EPI) formula.

| Sex | Scr (mg/dL) | Equation |
| --- | --- | --- |
| Female | ≤ 0.7 | eGFR =144(Scr/0.7)^-0.329^ × (0.993)^age^ |
|  | > 0.7 | eGFR =144(Scr/0.7)^-1.209^ × (0.993)^age^ |
| Male | ≤ 0.9 | eGFR =141(Scr/0.9)^-0.411^ × (0.993)^age^ |
|  | > 0.9 | eGFR =141(Scr/0.9)^-1.209^ × (0.993)^age^ |

Scr, serum creatinine; eGFR, estimated glomerular filtration rate
