# Supplementary material for: Association of renal hyperfiltration with incidence of dyslipidemia: a nationwide retrospective longitudinal cohort study
Source: PLoS One. 2025 Jun 3;20(6):e0324710. doi: 10.1371/journal.pone.0324710 (PMC12133170; doi:10.1371/journal.pone.0324710)
Supplement: S1 Table — (DOCX) [file pone.0324710.s003.docx]

**Supplementary table 1.** Definition of covariates.

| Diagnosis | ICD-10 code | Diagnostic definition |
| --- | --- | --- |
| Hypertension | At least one claim for I10-15 with the prescription of antihypertensive agents. | Systolic/diastolic blood pressure ≥ 140/90mmHg at baseline health examination or self-reported hypertension in the questionnaire. |
| Diabetes mellitus | At least one claim for E11-14ith the prescription of antidiabetic agents or two or more claims for E11-14. | Fasting glucose level ≥ 7.0 mmol/L at baseline health examination or self-reported diabetes mellitus in questionnaire. |
| Heart failure | The presence of I50 | At least one new patient and/or outpatient care for heart failure. |
| Myocardial infarction | At least one claim for I21-22 with the more than one hospitalization. | More than one hospitalization or baseline health examination or self-reported myocardial infarction in questionnaire. |
| Hyperthyroidism | The presence of E05 and two or more claims for E05 | baseline health examination or self-reported heart failure in questionnaire |

ICD, International Classification of Diseases.
