# Supplementary material for: Association of renal hyperfiltration with incidence of dyslipidemia: a nationwide retrospective longitudinal cohort study
Source: PLoS One. 2025 Jun 3;20(6):e0324710. doi: 10.1371/journal.pone.0324710 (PMC12133170; doi:10.1371/journal.pone.0324710)
Supplement: S2 Table — (DOCX) [file pone.0324710.s004.docx]

**Supplementary Table 2.** Comparison of baseline characteristics according to the incidence of dyslipidemia.

|  | Dyslipidemia (+)  (n=430,980) | Dyslipidemia (-)  (n=1,846,757) | P value |
| --- | --- | --- | --- |
| Sex |  |  | <.001 |
| Male | 215,579 (50.02) | 968,720 (52.46) |  |
| Female | 215,401 (49.98) | 878,037 (47.54) |  |
| Age, years | 54.03 ± 11.82 | 46.76 ± 14.38 | <.001 |
| Body mass index (kg/m^2^) | 24.42 ± 319 | 23.40 ± 3.24 | <.001 |
| Waist circumference (cm) | 82.22 ± 9.08 | 79.30 ± 9.37 | <.001 |
| Household income |  |  | <.001 |
| Q1, lowest | 116,689 (27.08) | 505,635 (27.38) |  |
| Q2 | 138,875 (32.22) | 661,676 (35.83) |  |
| Q3 | 113,186 (26.26) | 454,911 (24.63) |  |
| Q4, highest | 62,230 (14.44) | 224,535 (12.16) |  |
| Smoking status |  |  | <.001 |
| Never | 269,393 (62.51) | 1,132,335 (61.31) |  |
| Former | 66,457 (15.42) | 267,779 (14.50) |  |
| Current | 95,130 (22.07) | 446,643 (24.19) |  |
| Alcohol consumption  (days/week) |  |  | <.001 |
| None | 247,834 (57.50) | 952,147 (51.56) |  |
| 1–4 | 161,649 (37.51) | 825,984 (46.19) |  |
| ≥ 5 | 21,497 (4.99) | 68,626 (2.25) |  |
| Regular physical activity (days/week) |  |  | <.001 |
| None | 268,365 (62.27) | 1,122,990 (60.81) |  |
| 1–4 | 42,146 (9.78) | 175,329 (9.49) |  |
| ≥ 5 | 120,469 (23.78) | 548,438 (29.70) |  |
| Proteinuria |  |  | <.001 |
| Negative (-) | 408,410 (94.76) | 1,770,902 (95.90) |  |
| Positive (+) | 22,570 (5.24) | 75,855 (4.10) |  |
| Total cholesterol (mg/dL) | 199.93 (30.08) | 198.88 (28.64) | <.001 |
| Comorbidities |  | 1846757 |  |
| Diabetes mellitus | 43,443 (10.08) | 91,510 (4.96) | <.001 |
| Hypertension | 94,340 (21.89) | 221,330 (11.99) | <.001 |
| Heart failure | 5,836 (1.35) | 23,419 (1.27) | <.001 |
| Myocardial infarction | 559 (0.13) | 6,360 (0.34) | <.001 |
| Valvular heart disease | 1,196 (0.28) | 4,904 (0.27) | 0.171 |
| Cardiomyopathy | 332 (0.08) | 1,516 (0.08) | 0.294 |
| Hyperthyroidism | 4,888 (1.13) | 20,675 (1.12) | 0.412 |
| Congenital heart disease | 136 (0.03) | 678 (0.04) | 0.107 |
| Charlson comorbidity  index |  |  | <.001 |
| 0 | 233,153 (54.10) | 1,160,695 (62.85) |  |
| 1 | 116,822 (27.11) | 397,941 (21.55) |  |
| ≥ 2 | 81,005 (18.79) | 288,121 (15.60) |  |
| eGFR (decile),  mL/min/1.73 m^2^ |  |  | <.001 |
| 1st decile | 57.32 ± 7.61 | 59.85 ± 8.39 |  |
| 2nd decile | 69.23 ± 2.14 | 72.95 ± 2.20 |  |
| 3rd decile | 75.36 ± 1.47 | 79.43 ± 1.70 |  |
| 4th decile | 80.54 ± 1.57 | 85.04 ± 1.49 |  |
| 5th decile | 85.49 ± 1.27 | 89.65 ± 1.24 |  |
| 6th decile | 89.91 ± 1.33 | 94.29 ± 1.55 |  |
| 7th decile | 94.76 ± 1.48 | 99.52 ± 1.38 |  |
| 8th decile | 99.67 ± 1.35 | 104.54 ± 1.62 |  |
| 9th decile | 104.78 ± 1.67 | 111.20 ± 2.28 |  |
| 10th decile | 115.19 ± 10.77 | 122.81 ± 11.94 |  |
| eGFR (range),  mL/min/1.73 m^2^ |  |  | <.001 |
| < 60 | 22,508 (5.22) | 69,434 (3.76) |  |
| 60–89 | 216,801 (50.30) | 780,269 (42.25) |  |
| 90–120 | 184,843 (42.89) | 901,359 (48.81) |  |
| > 120 | 6,828 (1.59) | 95,695 (5.18) |  |

Data is presented as the mean ± standard deviation, or as a number (percentage).
Q, quartile; eGFR, estimated glomerular filtration rate.
