# Supplementary material for: Association of renal hyperfiltration with incidence of dyslipidemia: a nationwide retrospective longitudinal cohort study
Source: PLoS One. 2025 Jun 3;20(6):e0324710. doi: 10.1371/journal.pone.0324710 (PMC12133170; doi:10.1371/journal.pone.0324710)
Supplement: S9 Table — (DOCX) [file pone.0324710.s011.docx]

**Supplementary Table 9.** Association of renal hyperfiltration (range) with the incidence of dyslipidemia according to diabetes mellitus.

| Variable | DM | | | |
| --- | --- | --- | --- | --- |
|  | DM (+)  (n=134,953) | | DM (-)  (n=2,142,784) | |
|  | HR (95% CI) | P-value | HR (95% CI) | P-value |
| Sex |  | <.001 |  | <.001 |
| Male | Reference |  | Reference |  |
| Female | 1.06 (1.03, 1.09) |  | 1.27 (1.26, 1.28) |  |
| Age |  |  |  | <.001 |
| < 65 | Reference |  | Reference |  |
| ≥ 65 | 0.98 (0.96, 1.01) | 0.213 | 1.21 (1.20, 1.23) |  |
| Household income |  |  |  |  |
| Q1, lowest | Reference |  | Reference |  |
| Q2 | 0.99 (0.96, 1.01) | 0.239 | 0.93 (0.93, 0.94) | <.001 |
| Q3 | 1.02 (1.00, 1.05) | 0.075 | 1.02 (1.02, 1.03) | <.001 |
| Q4, highest | 0.98 (0.95, 1.01) | 0.246 | 1.09 (1.08, 1.10) | <.001 |
| Smoking status |  |  |  |  |
| Never | Reference |  | Reference |  |
| Former | 0.99 (0.96, 1.02) | 0.323 | 1.13 (1.12, 1.14) | <.001 |
| Current | 1.00 (0.98, 1.03) | 0.802 | 1.10 (1.09, 1.11) | <.001 |
| Alcohol consumption (days/week) |  |  |  | <.001 |
| None | Reference |  | Reference |  |
| 1-4 | 0.97 (0.95, 0.99) | 0.024 | 0.83 (0.82, 0.84) | <.001 |
| ≥ 5 | 1.06 (1.02, 1.10) | 0.003 | 1.14 (1.12, 1.15) | <.001 |
| Regular physical activity (days/week) |  |  |  |  |
| None | Reference |  | Reference |  |
| 1-4 | 0.90 (0.88, 0.94) | <.001 | 1.03 (1.02, 1.04) | <.001 |
| ≥ 5 | 0.96 (0.94, 0.98) | <.001 | 0.98 (0.97, 0.99) | <.001 |
| Body mass index (kg/m^2^) |  | 0.339 |  | <.001 |
| <25 | Reference |  | Reference |  |
| ≥25 | 1.01 (0.99, 1.03) |  | 1.32 (1.31, 1.33) |  |
| Waist circumference (cm) | 1.00 (1.00, 1.01) | <.001 | 1.01 (1.01, 1.01) | <.001 |
| Proteinuria |  |  |  |  |
| Negative (-) | Reference |  | Reference |  |
| Positive (+) | 1.12 (1.08, 1.15) | <.001 | 1.11 (1.10, 1.13) | <.001 |
| Total cholesterol (mg/dL) | 1.03 (1.01, 1.05) | <.001 | 1.02 (1.01, 1.05) | <.001 |
| Comorbidities |  |  |  |  |
| Hypertension | 1.11 (1.09, 1.13) | <.001 | 1.65 (1.63, 1.66) | <.001 |
| Heart failure | 0.73 (0.68, 0.78) | <.001 | 0.81 (0.79, 0.84) | <.001 |
| Myocardial infarction | 0.25 (0.20, 0.31) | <.001 | 0.34 (0.31, 0.37) | <.001 |
| Valvular heart disease | 0.75 (0.60, 0.92) | 0.006 | 0.96 (0.90, 1.02) | 0.152 |
| Cardiomyopathy | 0.72 (0.52, 0.99) | 0.043 | 0.87 (0.78, 0.98) | 0.018 |
| Hyperthyroidism | 0.68 (0.61, 0.75) | <.001 | 0.98 (0.95, 1.01) | 0.124 |
| Congenital heart disease | 0.30 (0.10, 0.93) | 0.037 | 0.93 (0.78, 1.10) | 0.398 |
| Charlson comorbidity index |  |  |  |  |
| 0 | Reference |  | Reference |  |
| 1 | 0.81 (0.79, 0.83) | <.001 | 1.28 (1.27, 1.29) | <.001 |
| ≥ 2 | 0.52 (0.50, 0.53) | <.001 | 1.14 (1.13, 1.15) | <.001 |
| eGFR, range, mL/min/1.73 m^2^ |  |  |  |  |
| < 60 | 0.81 (0.78, 0.84) | <.001 | 1.00 (0.99, 1.02) | 0.993 |
| 60-89 | Reference |  | Reference |  |
| 90-120 | 1.02 (1.00, 1.04) | 0.076 | 0.83 (0.83, 0.84) | <.001 |
| > 120 | 0.80 (0.74, 0.87) | <.001 | 0.34 (0.33, 0.35) | <.001 |

The multivariable model was adjusted for sex, age, income levels, smoking, alcohol consumption, regular physical activity body mass index, waist circumference, proteinuria, total cholesterol, hypertension, heart failure, myocardial infarction, valvular heart disease, cardiomyopathy, hyperthyroidism, congenital heart disease, Charlson comorbidity index and eGFR range.
DM, diabetes mellitus; HR, hazard ratio; CI, confidence interval; Q, quartile; eGFR, estimated glomerular filtration rate.
